# Practice on Metered Dose Inhaler Techniques and Its Associated Factors among Asthmatic Patients at Debre Markos Comprehensive Specialized Hospital, East Gojjam, Ethiopia: A Prospective Study

**DOI:** 10.1155/2021/6615727

**Published:** 2021-08-11

**Authors:** Tadele Asmare, Anteneh Belayneh, Bekalu Dessie

**Affiliations:** ^1^School of Medicine, Debre Markos University, Debre Markos, Ethiopia; ^2^Department of Pharmacy, College of Health Sciences, Debre Markos University, Debre Markos, Ethiopia

## Abstract

**Background:**

Asthma stands 16^th^ among the leading causes of years lived with disability and 28^th^ among the leading causes of disease in the world. *A metered-dose* inhaler remains the principal route for drug administration, and it has greater advantages over systemic treatment. In routine use, however, a majority of patients make inhalation errors. Suboptimal inhaler technique worsens health outcomes, with poor disease control, and increases the risk of hospitalization. This study aimed to assess practice metered-dose inhalation techniques and associated factors among asthmatic patients at Debre Markos Comprehensive Specialized Hospital, East Gojjam, Amhara region, Ethiopia.

**Methods:**

Prospective cross-sectional study was conducted from July 15 to August 30, 2020. Data were collected through a semistructured questionnaire. The data were analyzed using SPSS version 25. Associations between dependent and independent variables were assessed by using binary and multiple logistic regressions. *P* values less than 0.05 were considered to be statistically significant in all cases. Results are presented in tables, figures, numbers, and percentages.

**Result:**

A total of 166 patients had involved in the study, of which 52.4% were females. One hundred and eleven (66.9%) participants had good knowledge of asthma and inhalational techniques, while the rest of them had poor knowledge. One hundred and eight (65.1%) patients had effective practice on metered-dose inhaler use techniques. Participant's knowledge regarding asthma and meter dose inhaler and marital status has a significant association with their practice of metered-dose inhaler techniques with *P* value 0.001 and 0.006, respectively.

**Conclusion:**

In this study, most participants are suffering from asthma for a long duration and they have repeated exacerbation. Around two-thirds of patients had good knowledge regarding asthma and metered-dose inhaler and practice on metered-dose inhaler techniques. Participants with poor knowledge had poor practice on metered-dose inhaler techniques, and single patients were less likely to have poor practice on metered-dose inhaler techniques. Health education and counseling services should be consistently provided to the clients regarding the proper steps of inhalers use.

## 1. Introduction

Asthma is a persistent inflammatory disease of the airways in which many cells and cellular elements play a role that leads to recurrent episodes of wheezing, breathlessness, chest tightness, and coughing [1]. Asthma is ranked 16^th^ among the leading causes of years lived with disease and 28^th^ among the leading causes of disease in the world. The disease affects 1–18% population in the world [2].

Asthma continues to a global economic burden. In the USA, the total asthma costs increased from 53 billion US dollars in 2002 to 56 billion US dollars in 2011. Accurate estimates of the costs of treatments are not available for the majority of developing countries. Better disease management, improving access to health care, especially to preventer therapy, and improved adherence to such therapies significantly reduce the financial burden of the disease. Above 80% of asthma-related mortalities occur in developing countries [3–5].

Inhalation therapy forms are the mainstay of the treatment of asthma. Inhalation medications have fewer systemic side effects and immediate action than oral asthma medication. However, they need a skill that 70–80% of patients are unable to use inhaler correct metered-dose inhaler (MDI) which remains the principal route for drug administration, and it has greater advantages over systemic treatment. In routine use, however, a vast majority of patients make inhalation errors. Suboptimal inhaler technique worsens health outcomes, with poor disease control, and increases the risk of hospitalization. So, this study aims to know the cause of suboptimal usage and to assess the correct usage of MDI. Finally, it will improve the care and treatment of an asthmatic patient [1, 4, 6–8].

A cross-sectional study done in New Delhi, India, on 300 patients showed that 86% of patients identified their disease as breathlessness and 65% of them described it as repeated attacks of cough with or without expectoration [8]. In another cross-sectional survey conducted in Abu Dhabi, 67.7% of patients were confident about the use of inhalers. Almost half (50.4%) of parents were aware of an asthma action plan, and 42.5% knew the correct storage method for inhalers [9].

A descriptive study conducted among 105 patients in public hospitals in Sudan to assess knowledge and practice of asthmatic patients regarding the use of MDIs reflected the knowledge score of participants about steps of inhalation dose, steps of care postinhalation, storage, and clean the device, which are 77%, 44%, 79%, 43%, respectively, while participants had a poor level of knowledge about preparation of the dose, replacement, and cleaning mouthpiece with respective proportions of 63%, 68%, 60% [10].

A case-control study was conducted to evaluate determinants of uncontrolled asthma in Jimma University Medical Center on 121 controls and 121 cases which showed that 112 (92.6%) of the controls and 35 (28.9%) of the cases had good knowledge about asthma [11]. In another study done in Sudan, the level of participant's knowledge was very good regarding the care and storage of the device [12]. Most asthmatic patients cannot use their inhalers correctly. At least 50% of adults and children did not take controller medication as prescribed, and this will cause poor control of symptoms [13].

A prospective interventional study on knowledge, attitude, and practice towards MDI techniques conducted in India showed that, among 50 participants, 86.1% were using inhalers incorrectly. The common mistakes encountered on *g* inhaler techniques were as follows: not shaking the canister before use, unable to breathe hold, exhalation through the mouth, and not washing the mouth after using inhalers. In a similar study, 33% of the patients were not convinced about the diagnosis of asthma, and many had poor knowledge about inhalant therapy [14].

A study on 190 asthmatics having a follow-up at the department of pulmonary medicine done at Patiala College of Health Science showed that, about 74% preferred the use of inhalers for asthma. However, about 43% liked to use oral medications more than inhaled medicines. About 55% believed that inhaled medications contain a higher dose than oral (7).

An observational study done in India on an incorrect inhaler technique showed that, out of 89 patients using MDI, only 10 (11.2%) patients could demonstrate all the steps of inhaler usage correctly. 88.7% of patients had errors in one or more steps of the inhaler technique. The most common error was not holding the breath for 10 seconds which was seen in 46 (51.6%) patients. Thirty (33.7%) patients had an error of failure to exhale residual volume before inhalation. Three (3.3%) patients did not remove the cap from the canister and were unable to hold the inhaler upright [15].

The result of research done at different hospitals in Abu Dhabi also showed the attitude of patients towards asthma and their behavior regarding the use of inhalers and preventive therapy, and 57% of patients usually seek medical care for the treatment of asthma. 59% of asthma patients did not use anti-inflammatory drugs in the form of steroid inhalers when prescribed because they were afraid of lifelong dependence on inhalers. Most (76%) patients have felt safe taking their drugs when they were at work or away from home or outside of home [16].

A prospective study conducted among 140 patients on the MDI use technique at Jima University Specialized Hospital showed that most (92.85%) patients had errors in one or more steps of the inhaler technique. The commonest error was found to not inhaling slowly, simultaneously not pressing the canister, and failing to breathe in slowly and deeply which was committed by 69.28% of patients. The next frequent error was leaning head slightly back (49.3%) and taking the inhaler out of the mouth, and holding the breath for 5–10 sec was the third most missed step which accounts for 38.6% [17].

There are limited research findings to determine knowledge and practice towards inhalation techniques among asthmatic patients in Ethiopia, and specifically, there is no study at Debre Markos Comprehensive Specialized Hospital (DMCSH). There is repeated exacerbation of asthma in patients who have followed up at DMCSH who are on treatment. This study is, therefore, directed to investigate the level of practice MDI and associated factors among patients who have follow-up DMCSH.

## 2. Methodology

### 2.1. Study Area and Study Period

This study was conducted at DMCSH which is found in Debre Markos town. DMCSH has 4 major and 3 minor departments: medicine, surgery, pediatrics, GYN-obs, ophthalmology, psychiatry, and derma clinic. The medical department has also a ward, emergency, medical referral clinic, and 3 cold outpatient departments (OPD), emergency, and medical referral clinic [18].

The study was conducted from July 15, 2020, to August 30, 2020.

### 2.2. Study Design

A hospital-based cross-sectional study was conducted to evaluate practice towards metered-dose inhaler technique among asthmatic patients who visited DMCSH during the study period (from July 15, 2020, to August 30, 2020).

### 2.3. Source Population

The source populations for this study were all asthmatic patients who have a follow-up visit at DMCSH.

### 2.4. Study Population

The source populations for this study were all asthmatic patients who have follow-up visits at DMCSH who came during the study period.

### 2.5. Inclusion Criteria

Patients with known cases of asthma, with age above 18 years, who took inhalational medicine for at least 03 months, and who were willing to take part in the study, were enrolled for the study.

### 2.6. Exclusion Criteria

All asthmatic patients who are seriously ill during the data collection period and had other comorbid medical conditions were excluded from the study.

### 2.7. Sampling Technique and Sample Size

The sampling technique was convenient sampling. All cases who visited the hospital during the study period based on inclusion criteria were included in the study. The total sample size of the study was 166 participants.

### 2.8. Data Collection Tool and Method

Data were collected by using semistructured questionnaires prepared by adapting from different kinds of literature [7, 11, 14]. The questionnaire has three parts: sociodemographic characteristics, knowledge, and practices of asthmatic patients towards the use of metered-dose inhalers. The data were collected by the data collectors from the study participants. Demographic data were collected at the time of the interview, which includes age, gender, education status, occupation, area of residence, and frequency of inhaler use. A semistructured interview schedule regarding the use of inhalers is administered to collect the data. The interview questions were designed based on previous studies on this subject to assess the practice of the patients towards inhaler use. At the end of the interview, participants were allowed to share any of their concerns or opinions regarding inhaler use other than those mentioned in the proforma. Afterward, the interviewers spend time with the patient to educate them regarding any of their ill-knowledge or misconceptions about inhalation therapy.

### 2.9. Data Analysis

Data entry, coding, and analysis were performed using SPSS version 25 software package. Frequencies, percent, and summary statics were used to describe variables. Associations between dependent and independent variables were assessed by using binary and multiple logistic regressions. To measure internal consistency, reliability analysis was done. *P* values less than 0.05 were considered to be statistically significant in all cases.

### 2.10. Operational definition

Good knowledge: those respondents who answer 50% and above of knowledge questions.

Poor knowledge: those respondents who answer less than 50% of knowledge questions. There are 8 questions on the inhalation technique which were identified using a standard checklist of steps recommended by NIH guideline with one point given for each inhalational technique performed correctly with a maximum of a score being 8. Four or more correct responses are labeled as efficient, and the correct steps which are less than 4 were assigned as inefficient [14].

### 2.11. Dependent Variable

Respondent's practice on MDI was the dependent variable.

### 2.12. Independent Variables

Respondent's sociodemographic variables: age, sex, educational status, religion, residency duration of asthma, and knowledge towards MDI use were the independent variables of the study.

## 3. Result

### 3.1. Sociodemographic Characteristics

A total of 166 patients participated in the study, of which 87 (52.4%) were females and 79 (47.6%) were males. The average age of the participants was 41.10 years with a minimum age of 20 and a maximum age of 80 years. About 85 (51.2%) are residing in an urban area (Table 1).

### 3.2. Participants' Clinical Condition

From a total of 166 participants, the minimum and maximum duration of asthma were 7 and 70 years, respectively, with a mean duration of 27 years. Fourteen (8.4%) patients were suffering from asthma for a duration of less than or equal to ten years, and 76 (45.8%) are suffering from asthma for more than twenty-seven years with a maximum of 70 years (Table 2).

### 3.3. Knowledge about the Metered-Dose Inhaler Asthmatic Patient

**Figure 1:**
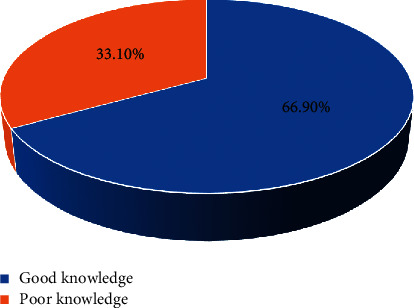
Knowledge regarding the disease and metered-dose inhaler, DMCSH, 2020.

All participants were aware of their diagnosis. The commonest side effect was fast heartbeat which has been reported by 119 (71.6%) patients, while 36 (21.7%) patients were complaining of shaking of hands. One hundred and fifty-six (94%) respondents used relievers in the time of acute exacerbation of asthma. One hundred and eleven (66.9%) participants had good knowledge, and 55 (33.1%) participants had poor knowledge regarding asthma and MDI (Figure 1).

### 3.4. Practice of Participant on MDI Use

All of the respondents believe that they can use the inhaler correctly. All participants were not using the refrigerator as storage. Most of them store their inhaler at room temperature away from direct sunshine exposure (Table 3).

Of 166 respondents, 108 (65.1%) patients were using the inhaler effectively, and the remaining 58 (34.9%) used the inhaler device ineffectively. The commonest mistake in using the inhaler was failing to check the label and expiry date (74%), not holding breath for 10 seconds (67.5%), and not breathing out gently (66.3%). Most participants practiced shaking well (79.5%) and replacing cap (72.3%) steps (Table 3).

### 3.5. Factors Associated with Practices towards MDI

In this study, the level of knowledge towards asthma and its management, residence, level of education, marital status, and occupation showed association with poor practice on bivariable logistic regression. But only knowledge and marital status showed a significant association on multivariable logistic regression. Patients with poor knowledge were 12 times more likely to have poor practice on MDI device use compared to those who had good knowledge (AOR = 12, 95% CI: 4.5, 31.9). Single patients were 0.8 times less likely to have poor practice on MDI compared to those who were widowed (AOR = 0.196, 95% CI: 0.05–0.8) (Table 4).

## 4. Discussion

Numerous studies on the knowledge and practices of patients regarding their illness are potent contributing factors to disease management. Asthma is a chronic disease in which a patient's good behaviors and practices are more significant in controlling the disease. The finding of this study revealed that 111 (66.9%) participants had good knowledge and 55 (33.1%) had poor knowledge on asthma and inhalational techniques. However, a case-control study done at Jimma University Medical Center reported 35 (28.9%) patients had good knowledge and 86 (71.5%) patients on the case had also poor knowledge [11]. This discrepancy might be due to the sociodemographic dissimilarities between the two populations, i.e., in our study, more than two-thirds of the patients are attending secondary school but in the study done at Jimma, and more than half of the participants were illiterate. In another study done at Jimma, 61.5% were efficient in using inhalers which is in line with our finding (95% CI: 57–71%) [17].

In our study, most of the patients believe that they can use the inhaler correctly, but only 108 (65.1%) patients use the inhaler effectively and the remaining 58 (34.9%) were using the inhaler ineffectively. The commonest mistake encountered was failing to check labels and expiration dates followed by not withholding breath for 10 seconds. Only 20 (12%) participants were performing all steps correctly. The study done in India on 200 participants showed that only 28 (14%) patients performed all steps correctly in use of inhaler devices and 172 (86%) patients were unable to use inhalers properly. The most common error for MDIs was being unable to hold breathe for 10 seconds which was seen in 46 (51.7%) patients. This difference could be explained by the sociodemographic and setups of the studies [15].

On multivariable regression, only marital status and level of knowledge had a significant association. Single patients were 0.8 times less likely to have poor practice on MDI compared to those who were widowed. Participants who had poor knowledge 12 times more likely to have poor MDI use practice than those with good knowledge. The study done at Jimma there showed a significant association between knowledge and practice [11].

## 5. Limitation of the study

There was a small sample size which is due to short period of data collection. The study sampling method was a nonprobability sampling method. So, it is difficult to generalize the study for all asthmatic patients. The study was based on self-reported information which is exposed to self-report bias. Moreover, this study did not consider the inhaler device from different vendors which may have an impact on the proper use of such device by the patients.

## 6. Conclusion

In this study, most participants are suffering from asthma for a long duration, and they have repeated exacerbation. Around two-thirds of patients have good knowledge, the rest have poor knowledge of asthma. More than half of the participants had good practice on MDI. Most participants who had poor knowledge had poor practice. Health education and counseling services should be consistently provided to the clients regarding the proper steps of inhalers use.

## Figures and Tables

**Table 1:** Sociodemographic characteristics of the respondents DMCSH, 2020.

		Sex		
		Male	Female	Total
Residency	Urban	36	49	85
	Rural	43	38	81

Age	18–40	32	47	79
	40–65	44	35	79
	≥65	3	5	8

Level of education	Unable to read and write	32	22	54
	Primary school	18	14	32
	Secondary school	12	17	29
	Diploma and above	17	34	51

	Total	79	87	166

Occupation	Government	9	19	28
	Private	21	44	65
	Others	49	24	73
	Total	79	87	166

Marital status	Single	18	1	19
	Married	49	44	93
	Divorced	1	6	7
	Widowed	11	36	47

	Total	79	87	166

**Table 2:** Duration of asthma of patients at DMCSH, 2020.

Duration of asthma	Sex		*N* (%)
	Male	Female	
≤10 years	5	9	14 (8.4)
10–27 years	32	44	76 (45.8)
>27 years	42	34	76 (45.8)
Total	79	87	166 (100)

**Table 3:** Practice of inhaler uses within the respondents, DMCSH, 2020.

No.	Questions	Yes	No
		*N* (%)	*N* (%)
1	Check the expiry date	43 (25.9)	123 (74.1)
2	Hold upright and shake well before use	132 (79.5)	34 (20.5)
3	Breath out gently	56 (33.7)	110 (66.3)
4	Put the mouthpiece between the teeth without biting and closed lips to form a good seal	158 (95.2)	8 (4.8)
5	Start to breathe in slowly through the mouth and press down firmly on the canister and continue to breathe in slowly and deep	144 (86.7)	22 (13.3)
6	Hold breath for 10 seconds and then remove inhaler from the mouth	54 (32.5)	112 (67.5)
7	Breath out gently	96 (57.8)	70 (42.2)
8	Replace the cap	120 (72.3)	46 (27.7)

**Table 4:** Factors associated with practices towards MDI use in DMCSH, 2020.

Variables	Practice		Cor, 95% CI	*P* value	AOR, 95% CI	*P* value
	Good	Poor				
*Sex*						
Male	51	28	1.00			
Female	57	30	0.959 (0.506, 1.816)	0.897		

*Marital Status*						
Single	13	6	0.4 (0.13–1.2)	0.116	0.196 (0.05–0.8)	**0.012**
Married	66	27	0.36 (0.174–0.74)	0.006	0.3	0.45
Divorced	7	0	0.000	0.99	0.00	0.00
Widowed	22	25	1			

*Occupation*						
Government	26	2	1			
Private	36	29	0.01 (0.694–2.7)	0.009	1.9	0.5
Others	46	27	0.13 (0.03–0.6)	0.01	1.75	

*Residency*						
Urban	65	20	1.00		1.00	
Rural	43	38	0.35 (0.18–0.7)	0.002	2.4 (0.7–7.8)	0.14

*Knowledge*						
Good knowledge	91	20	1.00		1.00	
Poor knowledge	17	38	10 (4.8, 21.5)	<0.001	12 (4.5, 31.9)	**<0.001**

## Data Availability

The data used for this study are available from the corresponding author upon request (bekiebda@gmail.com).
